# Samply: A user-friendly smartphone app and web-based means of scheduling and sending mobile notifications for experience-sampling research

**DOI:** 10.3758/s13428-020-01527-9

**Published:** 2021-02-02

**Authors:** Yury Shevchenko, Tim Kuhlmann, Ulf-Dietrich Reips

**Affiliations:** 1 Research Methods, Assessment, and iScience, Department of Psychology, University of Konstanz, Universitätsstraße 10, D-78464 Konstanz, Germany; 2 Differential Psychology, Assessment, and Research Methods, Department of Education Studies and Psychology, University of Siegen, Siegen, Germany

**Keywords:** Experience-sampling method, Mobile application, Notifications, Ecological momentary assessment

## Abstract

Undertaking an experience-sampling study via smartphones is complex. Scheduling and sending mobile notifications often requires the use of proprietary software that imposes limits on participants’ operating systems (whether iOS or Android) or the types of questions that can be asked via the application. We have developed an open-source platform—*Samply—*which overcomes these limitations. Researchers can access the entire interface via a browser, manage studies, schedule and send notifications linking to online surveys or experiments created in any Internet-based service or software, and monitor participants' responses—all without the coding skills usually needed to program a native mobile application. Participants can download the *Samply Research* mobile application for free from Google Play or the App Store, join a specific study, receive notifications and web links to surveys or experiments, and track their involvement. The mobile application leverages the power of the *React Native JavaScript* library, which allows it to be rendered in the native code of Android and iOS mobile operating systems. We describe *Samply*, provide a step-by-step example of conducting an experience-sampling study, and present the results of two validation studies. Study 1 demonstrates how we improved the website’s usability for researchers. Study 2 validates the mobile application’s data recording ability by analyzing a survey’s participation rate. The application’s possible limitations and how mobile device settings might affect its reliability are discussed.

## Introduction

The ways in which researchers have been able to observe participants have changed significantly over the years. When Barker and Wright (1951) turned the town of Oskaloosa in Kansas into their field laboratory and studied a boy’s life via continuous observation, there were no more advanced technologies than cameras and microphones to capture phenomena as they occurred in their natural settings. Continuous field observation was costly and burdensome, and a careful process was required to distill the full mass of information to its essence. Researchers thus turned to the idea of randomly sampling from the stream of experience. At the beginning of electronically supported experience-sampling research, participants had to carry pagers, and when those beeped, they wrote down details of their current experience. Smartphones have made it possible for participants to record their responses directly, making smartphone-based tasks equivalent to computer-based tasks. The question of whether smartphones can be used to bring experience sampling and other typically computer-based tasks from the laboratory to the field has already been answered positively in this journal with, e.g., supporting empirical evidence from our own laboratory (Stieger, Lewetz, & Reips, 2018).

Experience-sampling methodology (ESM) encompasses a range of methods based on prompting participants to respond to a survey or a task at various time points, which could be random (Ruwaard, Kooistra, & Thong, 2018). In the health sciences, this approach is known as *ecological momentary assessment* (EMA; Shiffman, Stone, & Hufford, 2008). The main idea is to assess peoples’ experiences or behaviors while they are having those experiences or exhibiting those behaviors, thus minimizing the potential for retrospective memory distortion (Schwarz & Sudman, 2012). A further advantage of ESM is increased external validity, as thoughts and behaviors are assessed in their natural environments (Stone, Shiffman, & DeVries, 1999).

Repeated assessments of varying intensity also enable a more detailed analysis of trends and processes than does cross-sectional research. Measurement intensities can be tailored to specific research questions and proposed processes. In addition to random interval sampling, with reminders sent by investigators, participants can also be asked to complete a survey when a specific event occurs—known as event-based sampling. For example, in a smoking cessation study by Shiffman et al. (2008), participants were asked to complete the survey every time they smoked a cigarette.

Numerous research questions can be answered using ESM data, such as individual differences, the influences of context and time, and interactions between personality and the environment (Shiffman et al., 2008). ESM is applicable not only in the health sciences but also in understanding human reactions to their environments: people’s thoughts and feelings can be used to create a representation of their environment, like a city. Research on *urban emotions* has examined people’s perceptions of and emotional reactions to city structures (Kohn et al., 2018; also see Klein & Reips, 2017) using data collected while they moved through them. Participants in Kohn et al.’s study (2018) reported their positive and negative emotions linked to locations in a park area. This approach can support the design and construction of optimized living places, where people are more satisfied with their surroundings. The present authors, for example, have been trying to cross-validate ESM ratings and enhance research about environments and users’ reactions to them by including data such as temperature, longitude, latitude, altitude, wind speed, rainfall, etc., from external databases to predict fluctuations in subjective ESM ratings (Stieger & Reips, 2019).

There is now a wide range of applications—commonly referred to as apps—supporting experience-sampling research (Conner, 2015). One approach developed in the late 1980s is based on Short Message Service (SMS) technology. Commercial companies like Twilio, Message Media, SurveySignal, or Poll Everywhere use SMS as part of their services (e.g., market research). This approach requires participants to send SMS messages when prompted (SMS-to-SMS technique). Since SMS must usually be paid for, the cost of messages is usually included in participants’ remuneration fees. Because messages are limited to 160 characters, commercial companies often send several questions in succession to elicit responses. To circumvent this limitation, some researchers gave participants paper-based forms with a coding scheme that allowed them to respond concisely with codes and abbreviations (Andrews, Bennett, & Drennan, 2011). Another way to overcome the limitations of SMS length is to provide a web link within the SMS which leads to an online survey. Hofmann and Patel (2015), for example, developed the web-based SurveySignal application, which links messages to online surveys.

An alternative approach is using a standalone application installed on a preprogrammed handheld device or the user’s smartphone (e.g., personal data assistants [PDA] vs. local application approach, in Hofmann & Patel, 2015). Participants using their smartphones must download and install an app to receive notifications, but as Hofmann and Patel (2015) noted, some participants may be wary of this procedure. Mistrust can be reduced if the app is distributed through official stores (Google Play or App Store), which indicates that the app has been reviewed and approved for the general public. Installing apps has since become common practice, and most smartphone users are experienced with the procedure.

Many of the proprietary standalone apps or web services for sending SMS have costs, either via a subscription model or a fee per participant or measurement. For example, the SurveySignal subscription plan costs USD 50 per month and is limited to 1000 “signals” (equivalent to 500 SMS messages); MovisensXS provides a standalone Android app and recommends an 8000-credit bundle (EUR 500) suited for a five-day survey involving 50 participants. Because *Samply* does not send SMS, but instead operates through a standalone smartphone app, there are no costs associated with notifications. *Samply* is free for both researchers and participants. Its open-source code is available via GitHub (https://github.com/Yury-Shevchenko/samply), and the web application for researchers is accessible at https://samply.js.org. We believe that the open-source approach will benefit *Samply*’s long-term sustainability (Bonaccorsi & Rossi, 2003).

Some standalone apps only work on one of the mobile platforms, such as only with iOS (ESm Capture, iDialogPad) or only with Android (MovisensXS, SampleMe, Aware). Our first approach also suffered from this limitation (Reips, Stieger, Heinrichs, & de Spindler, 2016), but the *Samply Research* mobile app is available on both major platforms and can be downloaded from Google Play for Android and App Store for iOS mobile operating systems. The original code (written with the *React Native JavaScript* library) is rendered in the native code on both platforms, so the application displays the same user interface as native applications. Because we maintain one codebase in JavaScript, the application is functionally identical on Android and iOS, with the only difference being the user interface’s platform-specific design.

Thirdly, even though experience-sampling software is available on both platforms, researchers are often limited to predefined question types. For example, ESm Capture only allows researchers to choose between binary, tripart, multiple choice, slider, and open-ended questions. The LifeData service has more options, but that app’s user interface limits customization. Instead, we have connected a mobile app to a mobile web browser by linking native push notifications to web links that are opened in the mobile browser. As a result, researchers can link browser-based studies to notifications sent by the *Samply Research* app. Many of today’s survey and experiment-building platforms (e.g., Qualtrics, SurveyMonkey, lab.js) are mobile-friendly. Responsive design adapts the user interface to the device. Another advantage of linking a web study to a mobile app is that no additional software needs to be installed on the participant’s smartphone: installing the *Samply Research* mobile app links the user to the web browser. Because our push notifications are sent using Internet protocols via Wi-Fi or cable and enable browser-based studies, our approach’s only prerequisite is the Internet connectivity of participants’ smartphones or other devices—SMS reception is not required, and devices that are not connected to a mobile service provider can also be used if they have an Internet connection. As the mobile Internet^1^ continues to expand (via phone providers or Wi-Fi networks), we believe that this approach is feasible.

The following work describes the methodological challenges of conducting an experience-sampling study and outlines the solutions already programmed in *Samply* (see Table 1). More technical details can be found on the project web page (https://samply.js.org). We subsequently provide a step-by-step tutorial for researchers interested in conducting a study using *Samply*, and finally, we present the results of two validation studies whose goals were to (1) improve the web app’s design and usability for researchers, and (2) validate the data collected through the *Samply Research* mobile app (i.e., participants’ interactions with notifications) by using an external survey’s results as a criterion.

**Table 1:** Overview of tasks and solutions implemented in *Samply*

Theme	Task	Solution in *Samply*
Content	Scheduling and sending notifications combined with the freedom to use any available tools to create a web page with a survey (e.g., Google Forms, Qualtrics, WEXTOR.eu, lab.js, jsPsych)	Customizable URL links sent to participants
Content	Informing participants about the survey	Customizable notification titles and message bodies
Device	Ensuring the compatibility of participants’ mobile devices	Support of compatibility tests
Schedule	Creating different notification schedules	Support of time- and interval-based schedules with an option to randomize notifications for each user
Analytics	Syncing survey data with information about how participants interact with notifications	Including a participant ID into the URL to record it in the survey; interactions with the notification are logged
Analytics	Improving participants’ compliance	Support of contingency plans (e.g., providing technical support to participants or changing the study format)
Data protection	Preserving participants’ anonymity	Participants do not need to provide researchers with any personal information like email addresses or phone numbers; all notifications are sent through the application

## Methodological challenges

### Notifications content

Notifications may cause disruption if they require attention at inopportune moments. Previous studies have shown that the sender–recipient relationship and the type, complexity and level of completion of the participant’s task interrupted by the notification all influence the response time and the perceived level of disruption (Mehrotra, Pejovic, Vermeulen, Hendley, & Musolesi, 2016). The notification’s priority level may also decrease the response time, and the higher the perceived level of disruption, the higher the probability of non-response.

Thus, the notification’s priority, suggested in the title and message, and the researcher–participant relationship can help to reduce the risk of notification dismissal. *Samply*’s notifications contain a title and a short message inviting the participant to click on it to complete a survey. In contrast to a text message that is limited to 160 characters, the *Samply* notification can contain an embedded web link of any length and unseen by the user. The notification’s three components of title, message, and study URL are customizable and can be changed in *Samply’s* administration menu by the researcher. The available length of the title and message displayed depends on the screen size and text language. Longer texts can appear trimmed on the smartphone screen (e.g., “Dear user, welcome to the survey on …”). In general, it is good to have a distinctive notification title, which helps participants quickly recognize that the notification is important. Further studies will be required to determine which notification-content features improve participants’ experience and reduce non-response.

### The effect of the mobile device

Van Berkel, Ferreira, and Kostakos (2017) found that about half of the studies in their meta-analysis provided participants with a mobile device, whereas participants in the other half used their own devices; the authors concluded that the use of personal devices in research was increasing. This trend is likely to continue as the number of personal mobile devices is growing. There are some disadvantages to providing participants with devices: they may have to learn how to use the new device, so some training may be required before the data collection, and they may feel uncomfortable about being observed by a dedicated device which they are unaccustomed to. Using homogeneous equipment across the study, however, gives researchers more control over how notifications and survey questions are displayed on the device (Meers, Dejonckheere, Kalokerinos, Rummens, & Kuppens, 2020).

The *Samply Research* native mobile app supports both strategies (providing a smartphone or having participants use personal ones) on both the Android and iOS mobile operating systems, which together accounted for 99.32% of market share worldwide in May 2020^2^.

Should the personal-device strategy be chosen, one way to ensure that the survey works on different users’ devices is to conduct a compatibility test (Hofmann & Patel, 2015). Participants are asked to respond to a notification, which takes them to a screening test where they provide information about their mobile device and practice answering questions in formats typical of those that will be used in the study. For example, they may be asked to select one option from a set of radio buttons, move a slider to set a specific value, or enter specified text in a text field. If their answers are correct, participants are given a password to proceed with the study.

### Notifications schedule

The other necessary component of an experience-sampling study app is the types of notification schedules it supports. In their meta-analysis, Van Berkel et al. (2017) found that the three most commonly used schedule types were randomized time points during the day (e.g., three times a day), time points at specific intervals (e.g., every hour), and event-dependent time points. *Samply* supports the first two types of schedule based on time points and intervals. Event-dependent notifications, as defined by the participant (e.g., smoking a cigarette), can be implemented via the *Samply Research* mobile app’s interface. The participant can be instructed to use the app to answer survey questions each time an event occurs (smoking a cigarette) . Notifications based on events detected by the mobile app using mobile sensors (GPS, gyroscope, etc.) are planned to be implemented in the future. For example, we are currently working on geofencing technology that will allow participants to be sent a notification when they enter or exit a specified location such as their home, office, or a public area (e.g., Klein & Reips, 2017; Nguyen et al., 2017).

*Samply* also records users’ interactions with their notifications, so it is possible to customize their notification schedule based on user response. For example, if compliance is low, researchers can implement a contingency plan such as contacting participants to help them with technical problems or switching to a paper-based version of the study (Thomas & Azmitia, 2016).

### Analytics

One line of research involves *interruption management*, i.e., analyzing people’s interactions with mobile notifications and exploring more efficient ways of interrupting users with notifications (Pielot et al., 2017). Switching between the phone’s vibrate, ring, and silent modes is one way that people often manage notifications. People rarely tend to adjust notification settings for individual apps, but usually prefer to change general settings (Pielot et al., 2017). Previous research found that 50% of interactions with notifications occurred in the first 30 seconds, and users’ most important notifications involve communication with other people (i.e., messengers) or provide information about the user’s context (i.e., calendar events) (Shirazi et al., 2014).

Ideally, information on users’ interactions with notifications should be available to researchers conducting experience-sampling studies. *Samply* automatically records the moments when notifications are sent from the server and when participants tap on them—information that can help researchers design better strategies for approaching participants.

One major practical question for a researcher is whether a notification should be withdrawn if it remains unanswered for some time. The push notifications used in *Samply* are displayed on the smartphone until the user interacts with them. Depending on the device, they may be hidden in the notification bar or shown in the background. It is up to the researcher to decide how to handle the data from participants (or their devices) who delay their responses. *Samply*’s analytics provide the data to make this decision: by comparing “sent” and “tapped” events in the notification history, the researcher can determine the length of time between the notification being displayed and the reaction to it.

### Data protection

Experience-sampling studies may contain personal data deserving protection, such as participants’ geolocation data. Because *Samply* does not collect survey data itself, only providing a venue for notifying participants, it is the researcher’s responsibility to ensure data confidentiality.

Participants must sign up to use the *Samply Research* mobile app with their email address. In this way, *Samply* prevents (to a certain degree) multiple participations in a single study on the same device. The email addresses are not shared with researchers and are only used for user authentication. Participants are not required to provide any demographic or sensitive information to use the app.

When a participant allows the app to send them notifications, a unique anonymous link is stored on the *Samply* server. This link is used to deliver notifications through iOS and Android services, and it contains no personal information about the user. When the participant leaves the study, the link is deleted from the server. *Samply* users have the option of contacting the researcher to request the deletion of their data (i.e., the log of interactions with notifications).

## Technological overview

*Samply* for researchers is a website at https://samply.uni-konstanz.de/ (or short samply.uni.kn) programmed in Node.js. The server side of the website is an Express.js server connected with a mongoDB database. Notification management is carried out through *cron*, a time-based job scheduler running on the server.

*Samply* for participants is a native mobile app written in the React Native JavaScript library and rendered with native code on Android and iOS. The mobile app uses push notification technology to deliver notifications.

## *Samply*: A step-by-step tutorial

### Researcher workflow

*Samply* is a web-based application that can be opened in any web browser (https://samply.uni.kn). In a nutshell, once a study notification plan has been created, a link is sent to the participants or is published online. Participants download the *Samply Research* mobile app, follow the link on their smartphones, sign up for the study, and start to receive notifications.

The key steps to using the web app as a researcher are:

1. Signing up
2. Creating a new study
3. Setting up notifications
4. Inviting participants
5. Monitoring participation

#### Signing up as a researcher

**Fig. 1:**
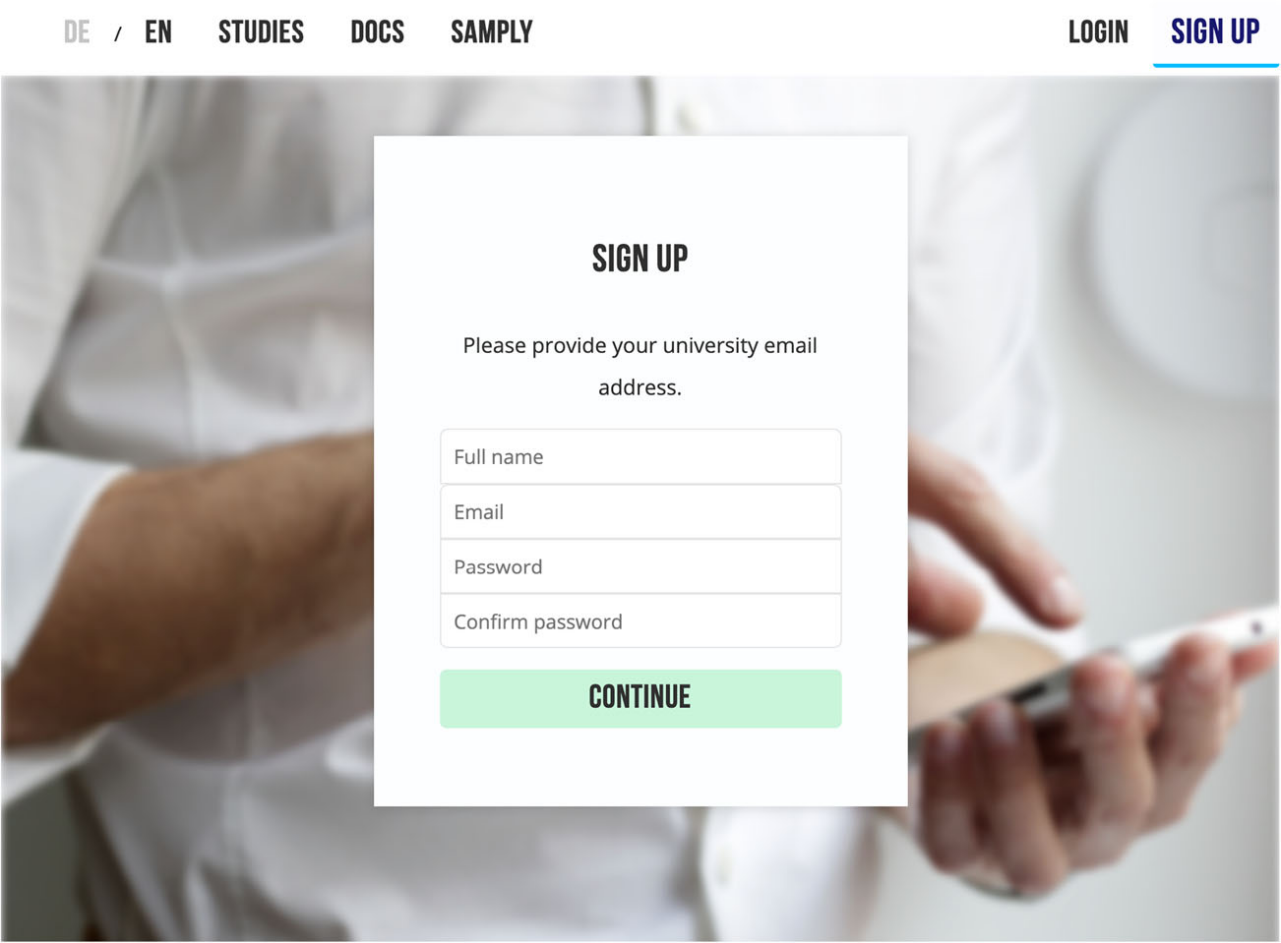
Screenshot of the Samply page showing the sign-up form for researchers

Signing up requires the researcher to enter an email address and create a password (Fig. 1). These will be used to log in to the account. If the password is forgotten, it can be reset via a link sent to the researcher by email.

#### Creating a new study

After the researcher signs up, they will be prompted to create a new study. They can write a study description to give their participants more information about what is expected of them in the study. The description will be displayed alongside the study’s name to help potential participants decide whether they wish to participate. A consent form text can also be shown to participants when they join the study. As an option, participants can be asked to enter a participation code or a username at this stage. Should a researcher wish to share access to the study with colleagues, their email addresses can be entered into the designated field (or left blank, as it can be done later). Colleagues should already be registered as researchers in *Samply* if they wish to have access to a study.

**Fig. 2:**
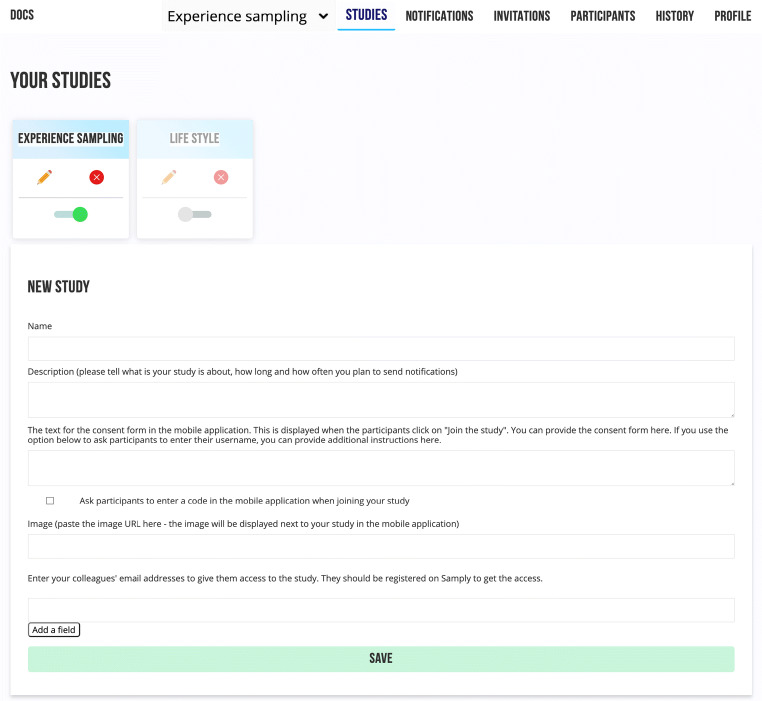
Screenshot of the page with the overview of the new study

After a new study has been added, a tab for it will be displayed on the web page (Fig. 2). The tab contains the study’s name and status along with two buttons to edit and delete it. The toggle switch at the bottom is grey when it is turned off—meaning that the study is not currently publicly accessible, i.e., not displayed in the list of studies in the mobile application. However, participants can still be invited using a direct link. When the study is ready to be opened to the public, the switch can be toggled to activate it. If a study has invited colleagues or registered participants, this information will also be displayed on the study tab.

#### Setting up notifications

Setting up the notifications plan requires defining the notification content (a title, message, and study URL) and deciding on the schedule. It is recommended that one keeps the title and message shorter than 30 characters each (including spaces) to make them compatible with all potential devices and screen sizes. The title can contain the study’s name (e.g., “Life style”) or the name of its current phase (“Life style – Day 1”). The message can include an instruction or what is expected from the participant, e.g., “Please complete a 1-minute survey.” Another strategy is placing a survey question in the notification title or message. For example, Pielot et al. (2017) put “How do you feel?” in the title and “Click to respond” in the message body.

The URL may contain the participant’s ID: the placeholder “%PARTICIPANT_CODE%” inside the web link will be replaced with each user’s *Samply* ID (e.g., https://examplesurvey.com/?id=%PARTICIPANT_CODE%). This can be used to track survey participation if the URL query parameters are captured and saved in the survey.

If the user interface is followed step-by-step, a few choices have to be made in order to define the notifications schedule.

1. Choose participants
  Notifications can be sent either to all current and future participants or to a particular participant chosen from the menu under “Current participants.”
2. Choose time(s)
  A specific time point or time points can be selected by entering the hour and minute in the input field. Alternatively, a time window can be defined (e.g., from 9 a.m. to 6 p.m.), during which a time point or time points can be randomly drawn. If several participants are selected, notification times will be randomized for each one.
3. Choose date(s)
  A specific date can be selected in the format of day, month, and year. Alternatively, if a notification needs to be repeated, other options can be chosen. Notifications can be sent every day or at a range of intervals (every 2nd, 3rd, 4th, etc. day). Alternatively, the day(s) of the week or day(s) of the month can be selected.
4. Choose month(s)
  Any specific month(s) can be chosen for notifications.
5. Choose when to start
  Notifications can begin to be sent at specific time points defined by the time and date. Alternatively, a time point can be picked relative to the current moment or the moment of participant registration. In the latter case, the starting point will be different for each participant and will be determined by the time when a participant joins the study via the mobile app.
6. Choose when to stop
  The options here are similar to choosing when to start notifications. Different combinations of starting and stopping points can be created; e.g., notifications can start from the moment each participant registers but stop at one specific time point, which will be the same for everybody.

When a notification has been added, it appears in the list of scheduled notifications, which can be controlled and cancelled at any time by clicking either the delete icon in the table or the “Delete all notifications” button.

#### Inviting participants

The “Invitations” page incorporates two ways of inviting participants to the study: a direct link to the study and using the search function inside the app. The direct link works even if the study is not publicly available in the list of studies. To use it, participants must already have the *Samply Research* app installed on their smartphone, and they should open the link in it too. When they open the app for the first time, a question about notifications appears automatically on the screen, and they must allow those notifications. Participants will also need to create a new account. Login information is stored securely on the *Samply* server (which is the University of Konstanz’s server, in Germany) and is only used to authenticate users.

After creating an account, participants are automatically redirected to the page within the app describing the study. When they click on the “Join the study” button, the consent form text appears, and on tapping the “Yes, I agree” button, they join the study and allow notifications from it. The application can then be closed.

Researchers can also add additional variables to the direct link using the query parameters; e.g., to create an individual link for a particular participant, the link can be modified by appending “&code=123” to the end of it. The value of the variable “*code*” (in this case it is *123*) will be recorded and shown in that particular participant’s data on the *Samply* website.

If, for some reason, participants cannot use the direct link, they can find the study in the list of studies within the app by using the search function. The study must be public, however. After logging in to the app, participants must click on the “Studies” tab and can then use the search field to find the study. The study’s name, author, and creation date are displayed in the list of studies. Tapping on the study transfers the participant to the study page.

#### Monitoring participation

**Fig. 3:**
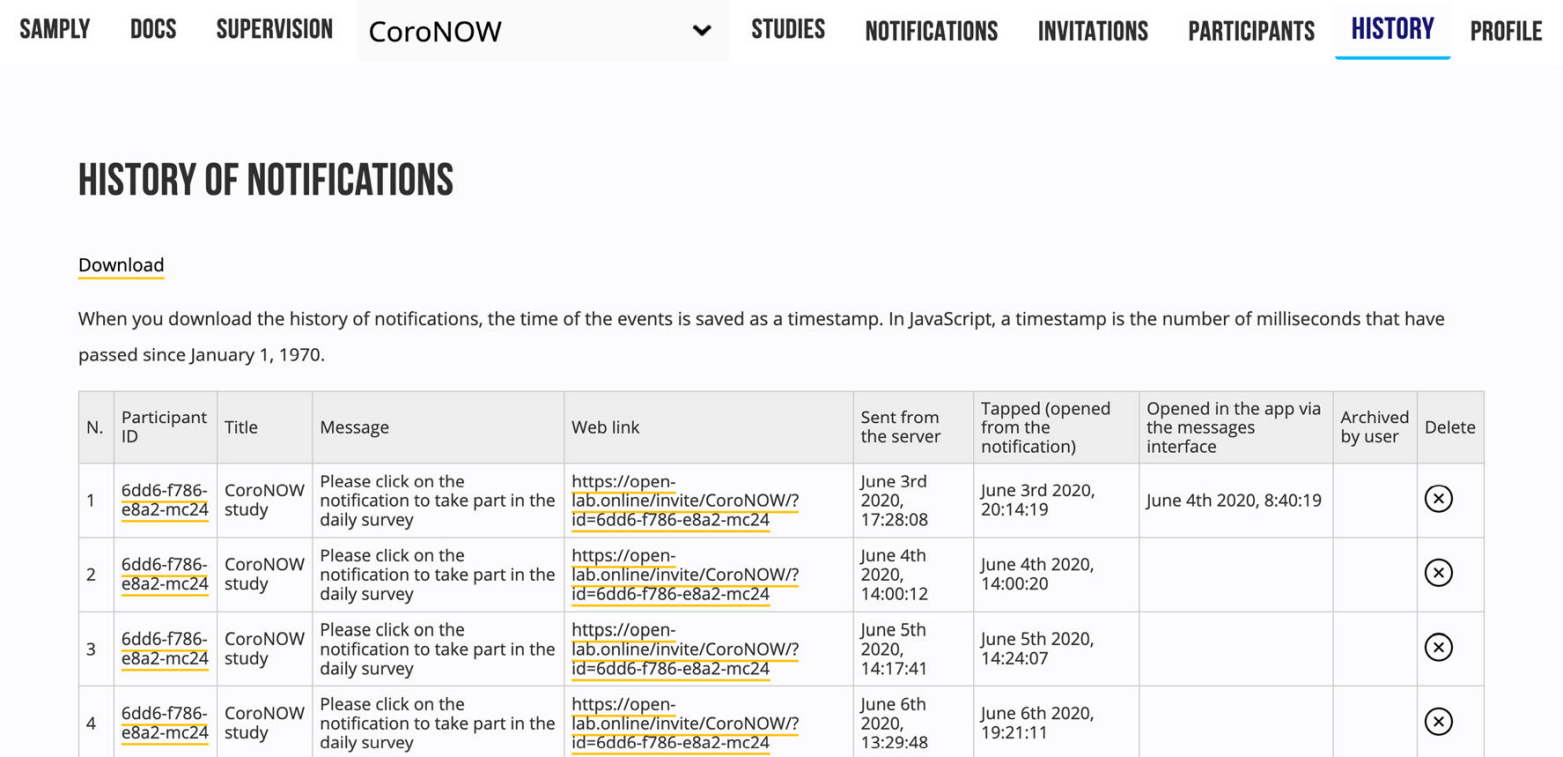
Screenshot of a page with an overview of a participant’s notifications

There are two ways of monitoring participation: via the list of participants (“Participants” menu) and the history of notifications sent (“History” menu). The list of participants displays them in order of registration to the study (Fig. 3). Together with their unique ID, the time at which they joined the study, and their username, the table provides information from the direct link query (e.g., code). The “Log” column displays the links to the logs of the notifications sent to a particular participant. Next to this, the “New notification” column has links to the Notifications menu where the participant’s notifications can be scheduled. A participant can also be deleted by clicking the basket icon on the right-hand side of the table.

The “History” menu displays the time points for the following events of each notification:

1. When the notification was sent from the server.
2. When the user opened the online study by tapping on the notification.
3. When the user opened the notification within the mobile application.
4. When the user archived the notification message within the mobile application.

The title, message, and study URL for each notification are shown. If the user dismisses a notification, no event is triggered or recorded. Clicking on the user ID reveals the notification events filtered for that user. To further inspect and analyze their interactions with notifications, the history of all the participants’ events can be downloaded in the CSV format.

#### Participant workflow

Once a participant has downloaded and installed the app on their smartphone, the study can be opened with the direct link shared by a researcher or found through the mobile app interface in the “Studies” tab. After joining the study, the participant can view it on their personal dashboard. The participant can later unsubscribe from the study by clicking the “Leave the study” button on the study’s page. All past notifications can be found in the “History” tab. The table displays the title, message, and study URL together with the time the notification was sent. A study link can be opened from the “History” tab by tapping on the notification. Participants can also customize the app by setting the times at which it is allowed to send them notifications. This information is displayed to the researcher on the page listing participants.

Next, we describe two validation studies. Study 1 aimed at improving the web app’s design and usability for researchers. Study 2 validated the data collected through the *Samply Research* mobile app by using an external survey’s results as a criterion.

## Empirical study 1

### Method

This first study’s goal was to test and improve the *Samply* website’s usability for researchers, and it was conducted from April to September 2020 with experience sampling method and usability experts. The experts evaluated the website’s functionality for researchers, particularly signing up, creating a study, and the notification scheduling interface.

#### Participants

We involved five usability experts and five researchers with expertise in experience-sampling methodologies, and we are grateful for their opinions on the website and app (see Acknowledgments).

#### Procedure

The usability study was conducted in two waves. Wave one (3 experts) took place from April to June, and wave two (7 experts) from June to September 2020. Experts were asked to contribute to the development of a free tool that would benefit the larger research community; they received no financial incentive to participate. Those who agreed received an instruction file with tasks to complete (see Appendix A) and installed the screencast software. The tasks were to sign up for a researcher account, create a study, schedule five different types of notifications, and edit the profile information. Participants could complete their tasks at their own pace, and they recorded screencasts as they completed them. Afterwards, they uploaded their screencast to the Dropbox link provided and completed the online usability survey.

The usability study was conducted in two waves to improve the website’s design and functionality based on the feedback received. Wave one participants were asked to participate in the usability study, and each one’s screen recordings and survey responses were analyzed. Based on wave one’s results, the website’s design and the user interface were substantially revised (see Table 4 for the revisions). Wave two participants were provided with the same new version of the website.

#### Materials

##### Instructions

The materials given to participants included explanations of the experience-sampling method and the *Samply* app, and step-by-step instructions on how to perform the usability test (see Appendix A). The instructions also contained a description of the screen recording software and the link to the online usability survey.

##### Usability survey

**Fig. 4:**
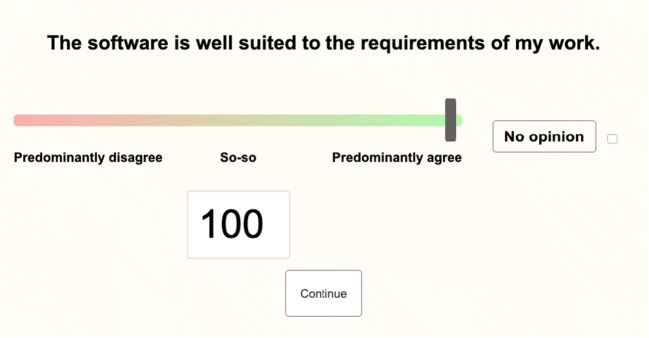
Screenshot of a page from the usability survey

The usability survey was based on the summative evaluation version of the IsoMetrics usability inventory (Gediga, Hamborg, & Düntsch, 1999). The IsoMetrics statements are usually grouped into seven scales that were derived using the principles of the ISO 9241-10 standard. We used 69 statements after removing items that were not relevant to the *Samply* website (e.g., “The software lets me change the names of commands, objects, and actions to suit my personal vocabulary.”) (see Table 5 for the statements). Participants were asked to rate each statement on a separate screen using a visual analogue scale from 0 (“Predominantly disagree”) to 100 (“Predominantly agree”) (see Fig. 4).

Participants were also asked whether they had encountered any errors or problems and how the app could be improved. The online survey was created in the lab.js experiment builder (https://lab.js.org, Henninger, Shevchenko, Mertens, Kieslich, & Hilbig, 2020) and hosted on the Open Lab platform^3^ (https://open-lab.online).

##### Screen recording

Instructions on how to use *QuickTime Player* for Mac and *Captura Portable* for Windows were provided in the task description for participants (see Appendix A). The recorded videos were uploaded to the first author’s Dropbox account using a public “upload file” link.

#### Analysis

The evaluation’s goal was to detect any usability problems within the app and improve its interface and design. The analysis of the screen recordings and the comments in the usability survey focused on three questions: (1) Were there any *critical problems* that interrupted the workflow? (2) Were there any *usability problems* that delayed the workflow? (3) What *possible improvements* could enhance the user experience?

The usability survey’s results were aggregated for each of the seven scales and are presented separately for wave one and two, as substantial changes were introduced between the two waves. Survey scales and items with a mean below 61 were considered to have a potential usability problem. This cutoff value was chosen in accordance with the four-point cutoff in the original IsoMetrics usability inventory. The analysis and visualization were done with R software, version 4.0.2 (R Core Team, 2020) and the ggplot2 package (Wickham, 2016). Data and materials are available at OSF (https://osf.io/u57aw/).

## Results

### Video analysis and revisions to the web application

Video analysis of wave one identified 11 critical problems and seven usability problems (see Table 2 for a summary and Table 4 for full information on the problems and app revisions). Eight of the critical problems were related to the use of the *cron* format in scheduling notifications, and the other three involved the display of scheduled notifications. Usability problems were due to the lack of an option to enter values using a keyboard, to missing or incorrect display of information, and to confusing error messages.

**Table 2:** Critical problems, usability problems, and potential improvements identified in waves one and two

Type	Summary of problems
Wave one	
Critical problems	1. Confusing notification schedule format (8 problems) 2. Uninformative display of scheduled notifications (3 problems)
Usability problems	1. Missing or confusing guidance information (3 problems) 2. Limited ability to enter values using a keyboard 3. Incorrect display on a small screen (2 problems) 4. Missing translations in the German language version (2 problems)
Wave two	
Usability problems	1. Confusions about the use of repeating schedules (6 problems) 2. Difficulty in finding some options (1 problem) 3. Error feedback (1 problem) 4. Selection of radio buttons
Potential improvements	1. Protection against potential errors when entering a colleague’s email address 2. Protection against the accidental deletion of scheduled notifications

During wave one, we revised the web application after both the first and second participant, fixing critical problems that had previously obstructed the user flow. Major changes were made after the third participant. First, *cron*-formatted strings were removed from the user interface so that researchers would not get confused by this new terminology. Instead, researchers were presented with step-by-step questions and choices between different scheduling options. For example, when the researcher chose the time of notification in the new interface, they had to select either a specific time point or a time window. Thus, the majority of critical problems, which might have prevented scheduling notifications, were eliminated. Second, we integrated critical information into the display of scheduled notifications. Each scheduled notification showed the settings chosen for each question asked during the planning stage. In this way, researchers could now spot mistakes (e.g., wrong choice of dates). Third, each usability problem was addressed by corresponding actions (see Table 4).

No critical problems were found in wave two, and the usability problems and potential improvements were addressed after the last participant.

### Survey

On average, experts chose the “No opinion” option for 15 (*SD* = 11) out of 69 items. The statement with the highest non-response rate was “I can adjust the presentation of results (on the screen, to printer, plotter, etc.) to my various work requirements,” which received a “No opinion” answer five times. Five statements (e.g., “I can call up specific explanations for the use of the system if necessary”) remained unanswered four times each.

**Fig. 5:**
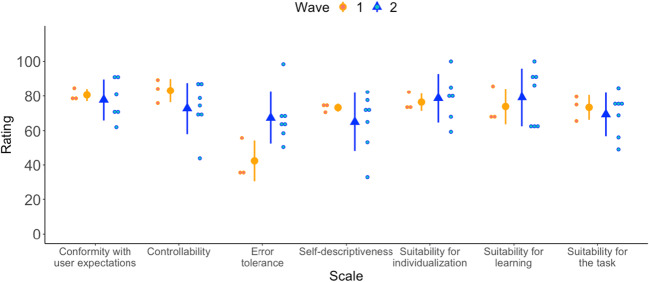
Average values of the usability study’s scales. *Note.* This figure shows the average values of the seven IsoMetrics scales for the groups of experts in waves one (*n* = 3) and two (*n* = 7). Error bars display one standard deviation above and below the mean. Small circles represent experts’ scores for each scale.

The mean scores for the seven IsoMetrics scales in waves one and two were all above the cutoff value of 61, except for the *Error tolerance* scale in wave one (*M* = 41.6, *SD* = 31.5). The means by scale and wave are displayed in Fig. 5. In wave one, the average rating across all scales was 71.9 (*Med* = 74.7, *SD* = 14.3), ranging from 34.3 to 89.3. In wave two, the average score was 72.6 (*Med* = 71.4, *SD* = 14.8), ranging from 33 to 100.

Some of the statements scoring below the cutoff value of 61, such as statement 35 (“When selecting menu items, I can speed things up by directly entering a letter or a command code”), could not have their underlying problem improved due to the website’s format. Other problems, however, such as statement 48 (“When I attempt to perform a destructive operation (e.g., data deletion, etc.), I am always asked to confirm the action.”), were addressed in the final revision of the program (see Table 4).

### Discussion

All the participants in this usability study were able to create an experience-sampling research study and use the scheduling tool for notifications. Wave one revealed critical errors that prevented some notifications from being saved correctly, but these were corrected in the subsequent app revision. The main improvement was changing the format from a *cron*-based string to a number of simple selection steps that created a schedule. We made the scheduler more flexible and improved error feedback, user control, and visibility of the system’s current state. All the participants in wave two were able to build the notification schedules specified in their instructions. There were no critical problems hindering system functionality. After wave two, usability problems were addressed, and potential improvements were implemented.

Data from surveying our expert participants confirmed the improved user experience between waves one and two, particularly in the application’s error tolerance. There remained some variability in the experts’ ratings, possibly due to different personal experiences with other online tools. Therefore, to improve the website’s general usability, we also added the documentation explaining each step of the researcher workflow. The notification planning page was enhanced with instructions and example schedules. In this way, the application now meets the needs of researchers with different experiences: it supports experienced users with error feedback and instructs newcomers with hints and clues. Finally, the experts’ comments in the usability survey were a source of new ideas and app enhancements.

*Samply* comprises two interlinked applications: the website for researchers and the mobile application for participants. The first study focused on improving the website’s usability for scheduling and planning notifications. In the second study, we evaluated the mobile application’s functionality for delivering notifications.

## Empirical study 2

### Method

The second study’s goal was to validate the *Samply Research* mobile application using the participation data from an external survey. The study was conducted in June and July 2020 in two student projects carried out during the experimental practicum at the University of Konstanz, Germany.

Because the study’s main goal was validation of the mobile app, we will omit a description of the underlying results obtained in the two student projects and instead focus on their participants’ response and dropout rates. The first project studied the effects of gratitude on well-being, whereas the second project examined the role of time management techniques in increasing the efficiency of studying. Participants were recruited via the University of Konstanz’s participant management system, SonaSystems, and through personal contacts with students.

#### Materials

##### The Samply Research mobile application

The *Samply Research* mobile app was available for free download from Google Play or the App Store. Participants in both projects completed the first browser-based survey online, receiving instructions on how to download and use the app. In order to match participants’ IDs in the Gratitude project, a random four-digit participant number was generated and displayed in the first survey together with the instructions for entering it in the mobile app. In the Time Management project, participants were asked to create and enter their own personal identification number (e.g., by using the initials) in both the online survey and the mobile app.

After the participants had installed the application and joined the projects, the scheduled notifications began. When participants tapped a notification in their smartphones, they were redirected to the first page of the mobile survey. The web link contained the participants’ *Samply* ID, so that each ID was recorded inside the survey and could later be matched with *Samply*’s data.

##### Web surveys and notification schedules

The projects’ web surveys were constructed in the lab.js experiment builder (https://lab.js.org, Henninger et al., 2020) and hosted on the Open Lab platform (https://open-lab.online).

The Gratitude project’s schedule sent notifications once a day for 14 consecutive days at a random time between 6 p.m. and 8 p.m. The notification contained a link to the project’s web survey that asked participants to list things they felt grateful for that day in one condition and things that had happened to them during the day in the control condition. Each daily survey took several minutes to complete.

The Time Management project’s schedule sent notifications once a day for 13 consecutive days at a random time between 6 p.m. and 8 p.m. The daily survey asked two questions, one on well-being and one rating personal studying progress.

The data and materials are available at OSF (https://osf.io/u57aw/), and both student projects were preregistered.

### Results

The *Samply Research* mobile app was installed by 76 participants, 44 of whom participated in the Gratitude project, 26 of whom joined the Time Management project, and 6 of whom participated in both. Thus, the Gratitude project collected data from 50 participants, and the Time Management project gathered data from 32. Eight participants failed to complete their daily surveys (*N*_*Gr*_ = 3 and *N*_*TM*_ = 5), so the final samples for the analysis consisted of 74 participants (*N*_*G*r_ = 47 and *N*_*T*M_ = 27) (see Table 3 for the participation rate).

**Table 3:** Student project’s participation rates

	Gratitude project	Time Management project	Total
Installed the mobile app	50 (100%)	32 (100%)	82 (100%)
Completed no surveys	3 (6%)	5 (16%)	8 (10%)
Completed at least one survey	47 (94%)	27 (84%)	74 (90%)
Completed at least 50% of surveys	38 (76%)	17 (53%)	55 (67%)
Completed at least 75% of surveys	27 (54%)	12 (38%)	39 (48%)
Completed every survey	4 (8%)	1 (3%)	5 (6%)

#### Compliance rate

The overall compliance rate, defined as the percentage of notifications that were followed by participation in a survey, was 65%, i.e., 657 out of 1011 notifications were followed (*Med* = 76.9, *SD* = 28.4).

**Fig. 6:**
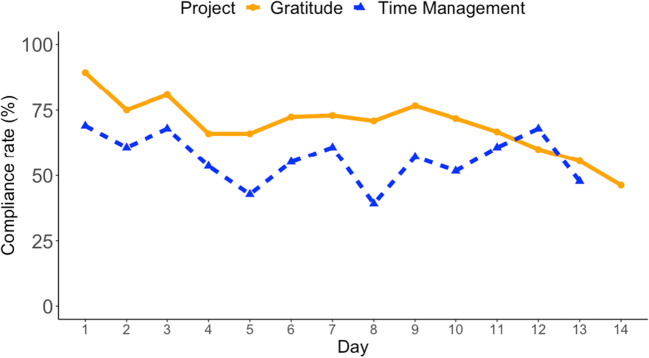
The compliance rates over the course of the two student projects. *Note.* The percentage of notifications (0–100%) that were followed by survey participation each day. The Gratitude project lasted 14 days and the Time Management project lasted 13 days.

To analyze the effects of the project and the participation day on the compliance rate, we applied a multiple linear regression model, using the participation rate (0–100%) as a dependent variable and project, participation day, and their interaction as predictors (see Fig. 6 for the compliance rate). The model explained a significant proportion of variance in the compliance rate, *R*^*2*^ = .58, *R*^*2*^_*adjusted*_ = .52, *F*(3, 23) = 10.37, *p* < .001. The main effect—which project was being participated in—was significant, indicating that the compliance rate in the Gratitude project, *M* = 69.8, *Med* = 71.3, *SD* = 10.7, was significantly higher than in the Time Management project, *M* = 56.1, *Med* = 57.1, *SD* = 9.4; *b* = −0.22, *t*(23) = −3.71, *p* = .001; unstandardized coefficients are reported. The main effect of participation day was also significant, showing that the compliance rate decreased by about 2% a day, *b* = −0.02, *t*(23) = −3.70, *p* = .01. There was no significant interaction between the project and the day, indicating that the decrease in participation over time was not significantly different between the two projects, *b* = 0.01, *t*(23) = 1.71, *p* = .10.

#### Response time

**Fig. 7:**
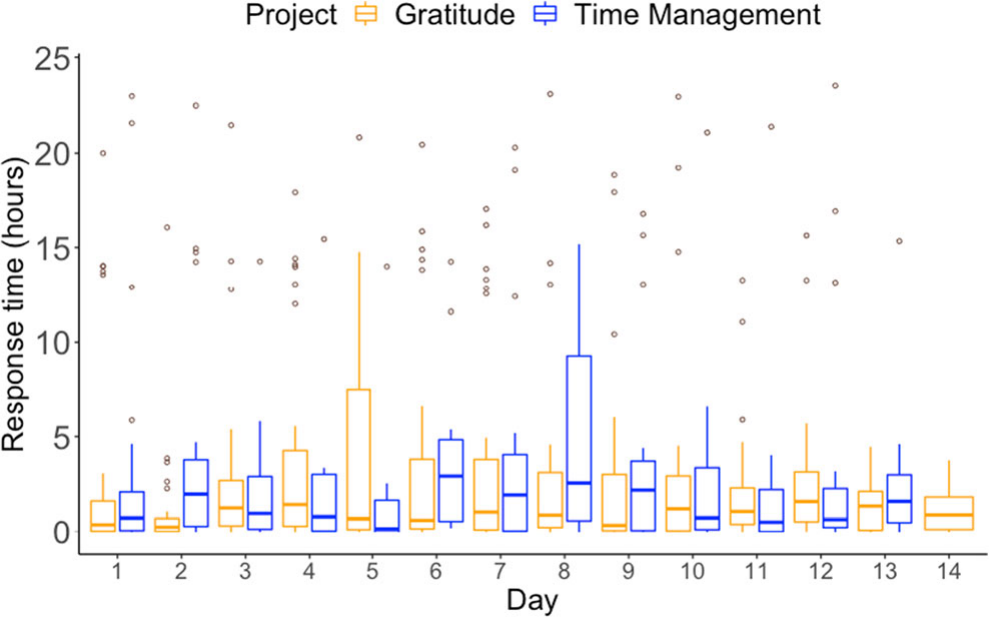
Response times across different participation days and projects. *Note.* Box plots display the response time medians (in hours) with the interquartile range (IQR) of the middle 50% of values. Whisker lengths are 1.5 × IQR. Outliers are represented by circles.

Study 2’s aim was to evaluate the *Samply Research* mobile app using participation data from student project surveys. Besides the compliance rate, another informative measure of an app’s usage is response time, which is defined as the time between when the notification is sent and when the survey web page is opened. Overall, the median response time across both projects was 52 minutes 34 seconds (in hours, *Med* = 0.9, *M* = 2.9, *SD* = 5.0). The response time was not significantly different between the Gratitude project, *Med* = 0.8, *M* = 2.7, *SD* = 4.6, and the Time Management project, *Med* = 1.1, *M* = 3.4, *SD* = 5.4, as confirmed by a two-sided Wilcoxon rank-sum test, *W* = 45346, *p* = .38. Response times did not change significantly over the course of the projects, as indicated by the day’s lack of any significant effect on the response time in a linear regression model, *b* = −0.03, *t*(660) = −0.62, *p* = .54, model fit: *R*^*2*^ < .001, *R*^*2*^_*adjusted*_ < .001, *F*(1, 660) = 0.38, *p* = .54 (see Fig. 7 for response times).

#### Interaction with notifications

The mobile app offered two ways to interact with notifications: tapping the notification in the smartphone’s notification bar or inside the mobile application. We analyzed the proportions and response times of interaction events to understand differences in the use of these two options. Of the 657 notifications that resulted in a survey response, 33% (*n* = 219) were opened by tapping the notification and 62% (*n* = 406) were opened from the app interface. Interestingly, 2.5% (*n* = 16) of notifications were both tapped and opened in the app interface, and 2.5% (*n* = 16) of the notifications had missing information about the user interaction^4^. Analyzing the same data by participants, we found that 39 participants (53%) both tapped and used the app to open the survey link, 11 (15%) only tapped, and 24 (32%) only used the app interface to open notifications.

The median time between when a notification was sent and when a participant tapped on it in the notification bar was 21 minutes 24 seconds (in hours, *Med* = 0.4, *M* = 1.9, *SD* = 4.1). The median time between when a notification was sent and when the survey link was opened in the mobile app was 1 hour 24 minutes 28 seconds (*Med* = 1.4, *M* = 6.8, *SD* = 25.6). The higher standard deviation in the latter case indicated the presence of outliers (in this case, participants who responded to the notification the next day).

#### Device effects

Based on the information recorded about their device when participants took part in the survey, 55% (*n* = 41) used Android smartphones and 45% (*n* = 34) used iOS. Comparing these participants’ percentage of notifications that were followed by participation in a survey (from 0 to 100%) using an independent two-sided *t* test, we found no significant differences between Android users (*M* = 66.3, *SD* = 29.9) and iOS users (*M* = 62.6, *SD* = 26.8), *t*(72.55) = 0.57, *p* = .57, *d* = −0.13, 95% CI [−0.59, 0.33]. There were also no significant differences in the individual median response times between groups of participants with Android, in hours *Med* = 0.9, *M* = 2.1, *SD* = 3.4, and iOS, *Med* = 1.8, *M* = 3.5, *SD* = 4.9, *t*(56.84) = −1.46, *p* = .15, *d* = 0.35, 95% CI [−0.11, 0.81].

### Discussion

#### Compliance rate

The second study’s goal was to validate the *Samply Research* mobile app’s feasibility with the participants of two student experience-sampling projects. We also used data from these external surveys to measure response times and compliance rates. The average compliance rate in our validation study (65%) was lower than the 75% rate reported in a meta-analysis of studies with more than six daily notifications (Wen, Schneider, Stone, & Spruijt-Metz, 2017) and was closer to the average compliance rate of 70% (SD = 23%) calculated in the meta-analysis by Van Berkel et al. (2017).

Interestingly, compliance rates differed between the two student project studies, which might have been due to their subject matter (one related to time management), the interactions between researchers and participants, or the sample characteristics (see meta-analysis in Vachon, Viechtbauer, Rintala, & Myin-Germeys, 2019). The Gratitude project, with its higher compliance rate, may have established a stronger connection between participants’ efforts and the intrinsic reward of self-reporting, which is a motivational factor acknowledged in previous research (Van Berkel et al., 2017). We also observed that compliance decreased with a sharp initial drop and then during the study at a rate of 2% a day, which was in line with observations of comparatively large dropout rates in online studies (Frick, Bächtiger, & Reips, 2001; Galesic, 2006; Reips, 2002, 2007).

#### Response time

The distribution of response times was highly skewed, with a median of around 52 minutes and outliers who only responded to notifications the next day. This could be considered an exceptionally long time interval but can be explained in light of the student project framework. Both projects sent one notification per day, with instructions stating that participants should provide their evaluation of that day (therefore notifications were sent in the evening). The instructions did not emphasize that participants should respond as fast as possible, although this type of instruction might be important for studies using a different schedule (e.g., several times a day). Furthermore, we did not let notifications expire, so the surveys could be opened at any time after receiving the notification. We used the same web link for these recurring surveys, but other studies could use different links to better monitor the survey’s progress.

Response times did not differ significantly between the projects and did not change throughout their durations, suggesting that participants who stayed with the projects retained the same pattern of application usage.

Participants in both projects made use of the possibility to open notifications via the app interface. Displaying notifications in the app was the backup plan to ensure that participants who had accidentally deleted a notification (e.g., by swiping it to the left instead of tapping it) could still find the survey link in the app. We did not explicitly instruct participants to use this feature, but more users did so (62%) than by tapping a notification (33%). Response times were significantly different, with notifications being tapped on after an average of 21 minutes or being opened via the application after an average of 1 hour 24 minutes. This may indicate that in cases where participants delayed their response, they preferred to use the app to get the link to the survey later. However, it could also be that they missed a notification and found it later in the app. The fact that most participants (67%) tapped a notification at least once suggests that this notification method did work for them technically. However, because participants were using their own devices, we could not prevent them from turning off notifications or switching to a battery-saving mode. Such concerns are generic for all mobile apps and should be discussed between researchers and participants at the beginning of a study.

#### Device effects

The participants in both student projects used their own Android or iOS smartphone systems. The absence of any significant differences in compliance rates and response times between the operating systems indicated that the *Samply Research* mobile app operates similarly on either device.

## General discussion

### Limitations

Researchers should be aware of the *Samply Research* mobile app’s two main limitations, but they are common to any mobile app that sends push notifications. First, participants need an Internet connection to receive notifications and open web links in the mobile browser. Secondly, participants must allow notifications in their phone settings. Depending on the model, additional steps may be required to enable notifications, and settings may be general or application-specific. For example, using the battery-saving mode may prevent the reception of notifications in Huawei phones. Moreover, if a participant uses task killer, battery-saver, performance-booster, task-cleaner, or security-type apps, these can also temporarily block notifications. If researchers have the opportunity to meet with participants at the beginning of a study, we recommend checking these settings with the participant so as to decrease the risk of undelivered notifications. In cases involving an Internet-based study, the *Samply* website has trouble-shooting instructions, which researchers can send to participants when inviting them to participate in a new study.

### Conclusion

*Samply* offers a number of advantages, including all the benefits of Internet services and open-source, web-based software. For researchers, the *Samply* platform makes organizing and conducting experience-sampling studies easier, without the need to program separate native mobile apps. Potential areas of use are Internet-based studies in which participants must respond more than once (e.g., diary studies, ecological momentary assessment). For participants, *Samply* is a convenient way to take part in research studies thanks to the convenience of using one’s own smartphone, e.g., as part of citizen science projects. The present paper provided a step-by-step tutorial on how to conduct a study using *Samply*, and it demonstrated the app’s functionality and usability via two empirical study projects organized by students. Our earlier usability study helped to significantly improve the *Samply* website’s ease of use for researchers, and our subsequent validation study confirmed the mobile app’s main functionalities. Further research should investigate the factors that influence participants’ responses to notifications, thus helping to develop more efficient strategies for interacting with them.

## Task Description in the Usability Study

### *Samply* Usability Study

Thank you very much for finding the time to participate in this usability study! In this study you will be asked to engage with the *Samply* website while recording your computer screen. Afterwards you will fill out a usability questionnaire and upload the video. Before you start with the usability test, please read the following information about the experience-sampling method and how *Samply* intends to support that method.

### Experience-Sampling Method

The experience-sampling methodology (ESM) is a range of methods based on prompting a participant to respond to a survey or a task at random time points throughout the day (Ruwaard et al., 2018). The main idea is to ask people to report their experiences or behavior exactly in the midst of their current activity. In this respect, this method should minimize memory distortions in comparison with retrospective memory. The advantage of ESM is an increase in external validity as thoughts and behavior are assessed in the natural environment.

### Samply

*Samply* makes sending notifications easy and provides a secure foundation for connection with participants. The application for researchers has an open-source code on GitHub and runs online at https://samply.uni-konstanz.de. The main idea is that researchers are free to use any tool to create an online survey or an experiment. *Samply* sends mobile notifications through the mobile application *Samply Research*, so when participants of the study click on the notification, they open the online study in a mobile web browser.

### Application features

- Native mobile application for participants available in Google Play or the App Store.
- Different types of notifications schedule (one-time, repeat, randomized).
- Customization of messages and URL links that are sent to participants.
- Preserving anonymity: participants do not need to provide any personal information such as emails or phone numbers. All notifications are sent through the application.
- Recording how participants interact with notifications.

### Instructions for the usability study

**Please follow the steps below:**

1. Download and install the screen recording software if required (please see the software instructions below on a separate page)
2. Start screen recording
3. Go to the website https://samply.uni-konstanz.de/
4. Create a new researcher account. Feel free to use your email address or choose a random email address (e.g. “*tester-{random number}@**gmail.com**”*).
5. After signing up, create a new study:
  - You can use an arbitrary name, description and consent form text for participants.
  - Share access to your study by entering the email address yury.shevchenko@uni.kn in the corresponding field.
6. Once you have created the study, edit its description. Edit it to any new text and save the changes.
7. Add two notifications with the following information:
  Title: Samply study
  Message: Fill in this survey
  Web link: https://iscience.uni-konstanz.de/
  Participants: All future participants
  1^st^ notification: Date and time: **October 1, 2020, at 15:00**
  2^nd^ notification: Date and time: **October 7, 2020, at 15:00**
8. Add a notification with the same title, message and web link, which should be sent to all future participants at a randomly chosen time between **12:00 and 18:00** on the **10**^**th**^ **of October, 2020**.
9. Add a new notification with the same title, message and web link, which should be sent to all future participants every day **at 15:00 from the 1**^**st**^ **of October to the 10**^**th**^ **of October, 2020.**
10. Add a new notification with the same title, message and web link, which should be sent to all future participants every day **at 9:30 for 5 days after user registration**.
11. Add a new notification with the same title, message and web link, which should be sent to all future participants **every second day between 09:00 and 12.00 for 10 days after user registration**.
12. Delete the notification scheduled for **October, 7 at 15:00**.
13. Proceed to the invitations tab and copy the direct link to your study. Paste the link to a text editing program. Later, paste this link into the usability questionnaire (see the step 18).
14. Go to your profile and update your profile information by entering your research institute or any random name (e.g. “University of Konstanz”).
15. Log out from the website application.
16. Stop recording
17. Upload the video at this link
  https://www.dropbox.com/request/q0pOpShzMvfxvvKZDYDD
  Use arbitrary names and email address, e.g.:
  First name *Joe*
  Last name *Dow*
  Email address joe.dow@gmail.com
18. Fill out the usability questionnaire at this link
  https://open-lab.online/code/Samply-Isometrics/?generate=true

### Screen recording software instructions

If you have a **Mac (for Windows see next page)**, we would recommend using QuickTime, because it is the **default** movie player for Mac and is usually already included in pre-installed software. If you don’t have QuickTime, you can download it from https://support.apple.com/downloads/quicktime

To create a new screen recording with QuickTime Player:

1. Launch QuickTime Player and choose **File, New Screen Recording** or use the shortcut **Shift + Cmd + 5** (available from macOS Mojave (version 10.14) onward)
2. Press the **Record** button. If there is a screen with additional instructions, choose **Start Recording**.
3. Complete the tasks on the Samply website.
4. When you are finished, click on **Stop Recording** on the Menu Bar. If you don’t see the menu, press **Command-Control-Escape** keys to stop the recording.
5. The video should be saved on the Desktop. If it is not saved, choose **File** and click **Save**. Select save location, then choose **Save**.

If you are lost, here you can find the instructions how to use QuickTime Player from Apple: https://support.apple.com/en-gb/guide/quicktime-player/qtp97b08e666/mac

If you use **Windows**, you can download Captura Portable (a free and open-source player) to make a screen recording. Download the portable version, which does not need to be installed from here https://mathewsachin.github.io/Captura/download/ or use the direct link: https://github.com/MathewSachin/Captura/releases/download/v8.0.0/Captura-Portable.zip

**Table 4:** Critical Problems, Usability Problems, and Potential Improvements Identified in Waves One and Two

Critical problems, usability problems, and possible improvements	Implemented solutions and improvements
First wave	
Critical problems	
General scheduling notifications interface: 1. One-time notifications could not be scheduled (the problem was specific to the web browser Opera) 2. Cron format of entering the schedule could be hard and confusing for users, could easily produce mistakes (e.g. by missing a symbol). 3. The beginning and ending time of the interval (in user-dependent random notifications) could be provided incorrectly – e.g. as 9.00 to 12.00 instead of 9.00 to 11.59 (since minutes and hours were independent). Users could be confused since there was no explanation provided on the page. (2 participants) 4. Wrong usage of * placeholder in the cron-formatted string ( *0 15* * * * instead of 0 15 * * * ) (2 participants) 5. The notifications were not saved in the list (server error at that moment) 6. The interface of interval picker is confusing – for users it is not clear that they should click twice to choose starting and ending date. Users use the option to type in the date with keyboard instead. 7. In Repeat notifications, the relationships between the time period and time interval are not clear. Users can try to manipulate the time period instead of setting up the time interval. 8. The scheduled notifications were displayed in the cron format, which could make a user unsure about the planned schedule (if the user could not interpret the cron format).	We completely redesigned the user interface for scheduling notifications. We removed cron-formatted strings from the user interface so a researcher does not get confused by this (possibly new) terminology. Instead, the researcher is presented with step-by-step questions and choices between different options. For example, when the researcher chooses the time of notification, she has to select either a specific time point or a time window. Thus, we eliminated the majority of critical problems that could prevent scheduling notifications in a correct way.
Display of scheduled notifications 1. No feedback whether the notification was correctly scheduled could cause critical errors (when the notification was not scheduled but the user did not know about that) 2. Planned notifications miss the information about time interval (in case of random notifications) 3. The information from scheduled notifications was hard to interpret	We integrated the critical information into the display of scheduled notifications. Each scheduled notification shows the chosen settings for each question asked during the planning stage. In this case, a researcher can spot a mistake if it happened (e.g., wrong choice of dates).
Usability problems	
The placeholder “Confirm password” was not displayed correctly on the sign up page.	The placeholder “Confirm password” is displayed in the password input field.
It was not possible to manually enter the time with a keyboard so the user had to click through the time picker buttons.	In the new interface it’s possible to enter time values directly using a keyboard.
The display of time interval picker in the table was confusing on the small screen	We replaced the time interval picker with simple input fields that are displayed correctly on screens of different sizes.
The display of scheduled notifications in the table was confusing on the small screen.	We implemented responsive design to adjust the display format to the user screen size.
The meaning of event-dependent notifications was not clear	We removed the reference to event-dependent notification, which was misleading in the old user interface. Instead, in the new interface, a researcher can choose to start and end notifications dependent on the moment of the participant registration.
Confusing message “Sorry, notifications are not possible on your device, if you want to take part in a study”. This message is for participants, but researchers also see that.	This message was removed in the new version of the application, which now supports both iOS and Android systems.
Missing translations in German version - FAQ titles were displayed in English in German version of the website. - In the German version some of the buttons had English names	The buttons and titles were translated into German.
Potential improvements	
No possible improvements could be combined in a separate category, since majority of improvements were related to the usability and critical problems.	
Second wave	
Critical problems	
No critical problems were found that interfere with the functionality of the application.	
Usability problems	
Confusion with the notification planner interface: - The user was confused when it came to scheduling the notification between September 1 and 10. The user chose the start and end dates, but not the month and not the time of notification. - The user confused the time and date when creating two notifications for the same time but different days. The time was entered twice. - The user forgot to select the month.	Supplementary help information has been provided on the webpage.
It was easy to miss the opportunity to start/stop sending notifications from the moment of participant registration.	We have added the time of participant registration as the first default option in the drop down menu.
If the user made a mistake when scheduling the date/time, the system did not highlight the wrong sections that prevented the user from completing the task.	The information about missing entries was provided in warning messages.
If the user chose a specific month (e.g. September), the whole category "Specific month(s)" was not selected.	The response category should be selected automatically when the user selects a subcategory.
The "When to start/end" section might be confusing - a user has tried to select the exact time of notification as the start moment.	This option was secured by adding a 1-minute buffer before and after the end of the notification schedule.
In the case of relative notification, the user mistakenly chose September only (but it should be any month, as the schedule can start at any time).	Supplementary help information has been provided next to the “Choose month” section.
The user missed the instruction to send a notification every other day (instead she chose every day).	Made the more important information more salient in the display of scheduled notifications.
What happens if the notifications are scheduled for the time already passed?	Show a warning message in this case.
Potential improvements	
Creating study page: The email of a colleague might contain a space around it – in this case, it would not be found in the database.	Trim the email address before searching for it in the database.
The scheduled notification could be accidentally deleted.	Added a dialog to confirm deletion of a notification.
It was inconvenient to re-enter the same information for title and message if the user scheduled many notifications.	Keep the input text in the input fields.
On the page with studies, it might be hard to understand the location of edit, delete, publish buttons on the study card.	Display the information message when a user moves the mouse over the study card.
The link about Query string leads to the English source in the German version (Notifications page).	Put the German link about Query string into the German version.
No option to withdraw from deleting a study and getting back to the previous window.	Added the button “Cancel” that returns to the page with studies.
The user suggested changing the button name to “Schedule notification(s)”	Changed the button to “Schedule notification(s)”
Translations: - Translate “please provide your university email address” and placeholders into German for German version on “register” page. The same is for login page (placeholders should be translated into German) - Alert messages should be translated into German (for example, “Choose the month”)	Translations were done.
The specific date appears after other options inside of the rubric “Choose the date” – some people might not notice it.	Display the specific date input field right next to the option “Specific date”.
New study page – users might want to click on “Add a field” when adding a collaborator.	Instead of “add a field” changed to “add a new empty field” (to make clear that it’s about adding a new empty field for the email address).
The sign up form takes the width of whole screen on Mozilla Firefox	Adjusted the form size
Enhancing feature	Added option to send immediate notification (for testing the functionality of the mobile app)

## The Statements of the Usability Survey

**Table 5:** The statements were adapted from the summative evaluation version of IsoMetrics usability inventory (Gediga et al., 1999)

Number	Statement	Scale
1	The software forces me to perform tasks that are not related to my actual work.	Suitability for the task
2	The software lets me completely perform entire work routines.	Suitability for the task
3	The functions implemented in the software support me in performing my work.	Suitability for the task
4	The way in which data is entered is suited to the tasks I want to perform with the software.	Suitability for the task
5	I perceive the arrangement of fields on-screen as sensible for the work I do with the software.	Suitability for the task
6	Too many different steps need to be performed to deal with the given task.	Suitability for the task
7	The way in which data is output (e.g. the table of scheduled notifications) is suited to the tasks I want to perform with the software.	Suitability for the task
8	The software is well suited to the requirements of my work.	Suitability for the task
9	In a given screen, I find all of the information I need in that situation.	Suitability for the task
10	The terminology used in the software reflects that of my work environment.	Suitability for the task
11	The software provides me with a repeat function for work steps that must be performed several times in succession.	Suitability for the task
12	The important commands required to perform my work are easy to find.	Suitability for the task
13	I am able to adjust the presentation of results (on the screen, to printer, plotter, etc.) to my various work requirements.	Suitability for the task
14	The presentation of the information on the screen supports me in performing my work.	Suitability for the task
15	I can easily adapt the software for performing new tasks.	Suitability for the task
16	I can call up specific explanations for the use of the system if necessary.	Self-descriptiveness
17	I understand immediately what is meant by messages displayed by the software.	Self-descriptiveness
18	It is easy to retrieve information about a certain entry field.	Self-descriptiveness
19	When menu items are not available in certain situations, this fact is visually communicated to me.	Self-descriptiveness
20	If I want, the software will display not only general explanations but also concrete examples to illustrate points.	Self-descriptiveness
21	The explanations the software gives me clearly refer to the specific situations in which they are output.	Self-descriptiveness
22	If I want, the software provides me with enough information about which entries are permitted in a particular situation.	Self-descriptiveness
23	I can tell straight away which functions are invoked by the various menu items.	Self-descriptiveness
24	The terms and concepts used in the software are clear and unambiguous.	Self-descriptiveness
25	The software visually marks the current entry location (e.g. by a highlighting, a contrasting color, a blinking cursor, etc.).	Self-descriptiveness
26	I can easily tell the difference among feedback messages, requests to confirm inputs or commands, warnings, and error messages.	Self-descriptiveness
27	The possiblilities for navigating within the software are adequate.	Controllability
28	The software makes it easy for me to switch between different menu levels.	Controllability
29	The software lets me return directly to the main menu from any screen.	Controllability
30	I can interrupt any dialog at any time.	Controllability
31	It is always easy for me to move back and forth between different screens.	Controllability
32	The software allows me to interrupt functions at any point, even if it is waiting for me to make an entry.	Controllability
33	The navigation facilities of the software support optimal usage of the system functionality.	Controllability
34	In order to perform my tasks, the software requires me to perform a fixed sequence of steps.	Controllability
35	When selecting menu items, I can speed things up by directly entering a letter or a command code.	Controllability
36	It is always possible to abort a running procedure manually.	Controllability
37	The software is inconsistently designed, thus making it more difficult for me to do my work.	Conformity with user expectations
38	I can anticipate which screen will appear next in a processing sequence.	Conformity with user expectations
39	I have no difficulty in predicting how long the software will need to perform a given task.	Conformity with user expectations
40	The designations are used consistently in all parts of the software I am familiar with.	Conformity with user expectations
41	I find that the same function keys are used throughout the program for the same functions.	Conformity with user expectations
42	When executing functions, I have the feeling that the results are predictable.	Conformity with user expectations
43	My impression is that the same possibilities are consistently available for moving within and between different parts of the software.	Conformity with user expectations
44	The messages output by the software always appear in the same screen location.	Conformity with user expectations
45	When working with the software, even small mistakes have sometimes had serious consequences.	Error tolerance
46	Even if I make a mistake, the information (e.g. data, text, and graphics) which I have just entered is not lost.	Error tolerance
47	If I make mistake while completing a form, I can easily restore everything to its previous state.	Error tolerance
48	When I attempt to perform a destructive operation (e.g. deletion of data, etc.), I am always first prompted to confirm the action.	Error tolerance
49	My impression is that very little effort is involved in correcting mistakes.	Error tolerance
50	When I make entries, they are first checked for correctness before further processing is initiated.	Error tolerance
51	No system errors (e.g. crashes) occur when I work with the software.	Error tolerance
52	If I make a mistake while performing a task, I can easily undo the last operation.	Error tolerance
53	I have never made an entry that caused a software error (e.g. a system/program crash or an undefined dialog state).	Error tolerance
54	The software includes safety features to help prevent unintended actions (e.g. critical keys spaced well apart, highlights, designations that are not easily confused).	Error tolerance
55	The software provides me with useful information on how to recover from error situations.	Error tolerance
56	I perceive the error messages as helpful.	Error tolerance
57	In some situations the software waits too long before calling attention to wrong entries.	Error tolerance
58	The software warns me about potential problem situations.	Error tolerance
59	The software lets me keep the original data after it has been changed.	Error tolerance
60	The software can be easily adapted to suit my own level of knowledge and skill.	Suitability for individualization
61	I can adjust the notification schedule to suit my individual needs.	Suitability for individualization
62	I can adjust the attributes of the software to my research needs.	Suitability for individualization
63	I can adjust the amount of information (data, text, graphics, etc.) displayed on-screen to my needs.	Suitability for individualization
64	I needed a long time to learn how to use the software.	Suitability for learning
65	It will be easy for me to relearn how to use the software after a lengthy interruption.	Suitability for learning
66	The explanations provided help me understand the software so that I become more and more skilled at using it.	Suitability for learning
67	So far I have not had any problems in learning the rules for communicating with the software, i.e. data entry.	Suitability for learning
68	I would be able to use the software right from the beginning by myself, without having to ask other people (or technical support) for help.	Suitability for learning
69	I feel encouraged by the software to try out new system functions by trial and error.	Suitability for learning

## Footnotes

1 The number of mobile broadband subscriptions has increased over the years (retrieved on 13.07.2020) https://data.oecd.org/broadband/mobile-broadband-subscriptions.htm

2 Statcounter Global Stats (retrieved on 25.06.2020) https://gs.statcounter.com/os-market-share/mobile/worldwide

3 The usability survey is available at https://open-lab.online/tests/test/isometrics-usability

4 The missing information error was later corrected in the updated version of the *Samply Research* mobile app.
